# Recent range expansion of an intermediate host for animal schistosome parasites in the Indo-Australian Archipelago: phylogeography of the freshwater gastropod *Indoplanorbis exustus* in South and Southeast Asia

**DOI:** 10.1186/s13071-017-2043-6

**Published:** 2017-03-06

**Authors:** Pauline Gauffre-Autelin, Thomas von Rintelen, Björn Stelbrink, Christian Albrecht

**Affiliations:** 1 Department of Animal Ecology and Systematics, Justus Liebig University, Giessen, Germany; 2 Museum für Naturkunde, Leibniz Institute for Evolution and Biodiversity Science, Berlin, Germany

**Keywords:** Historical phylogeography, Indo-Malaya, Cryptic species complex, *Schistosoma indicum* species group, Neglected tropical disease

## Abstract

**Background:**

The planorbid snail *Indoplanorbis exustus* is the sole intermediate host for the *Schistosoma indicum* species group, trematode parasites responsible for cattle schistosomiasis and human cercarial dermatitis. This freshwater snail is widely distributed in Southern Asia, ranging from Iran to China eastwards including India and from the southeastern Himalayas to Southeast Asia southwards. The veterinary and medical importance of this snail explains the interest in understanding its geographical distribution patterns and evolutionary history. In this study, we used a large and comprehensive sampling throughout Indo-Malaya, including specimens from South India and Indonesia, areas that have been formerly less studied.

**Results:**

The phylogenetic inference revealed five highly divergent clades (genetic distances among clades: 4.4–13.9%) that are morphologically indistinguishable, supporting the assumption that this presumed nominal species may represent a cryptic species complex. The species group may have originated in the humid subtropical plains of Nepal or in southern adjacent regions in the Early Miocene. The major cladogenetic events leading to the fives clades occurred successively from the Early Miocene to the Early Pleistocene, coinciding with major periods of monsoonal intensification associated with major regional paleogeographic events in the Miocene and repeated climate changes due to the Plio-Pleistocene climatic oscillations. Our coverage of the Indo-Australian Archipelago (IAA) highlights the presence of a single clade there. Contrary to expectations, an AMOVA did not reveal any population genetic structure among islands or along a widely recognised zoogeographical regional barrier, suggesting a recent colonisation independent of natural biogeographical constraints. Neutrality tests and mismatch distributions suggested a sudden demographic and spatial population expansion that could have occurred naturally in the Pleistocene or may possibly result of a modern colonisation triggered by anthropogenic activities.

**Conclusions:**

Even though *Indoplanorbis* is the main focus of this study, our findings may also have important implications for fully understanding its role in hosting digenetic trematodes. The existence of a cryptic species complex, the historical phylogeographical patterns and the recent range expansion in the IAA provide meaningful insights to the understanding and monitoring of the parasites potential spread. It brings a substantial contribution to veterinary and public health issues.

**Electronic supplementary material:**

The online version of this article (doi:10.1186/s13071-017-2043-6) contains supplementary material, which is available to authorized users.

## Background

The trematode genus *Schistosoma* Weinland, 1858 (Strigeiformes: Schistosomatidae) represents parasitic flatworms affecting mammals including humans, causing hepato-intestinal, urogenital, pulmonary and nasal schistosomiasis. Since these parasites use freshwater snails as intermediate hosts for their larval development, they are also responsible for cercarial dermatitis in human populations that are in contact with infested waters [1, 2]. Because of their considerable medical and veterinary importance, a full understanding of the mechanisms underlying the distribution and successful spread of these parasites becomes paramount for effective control and prevention.

Amongst the four recognised common *Schistosoma* species groups [3], the *Schistosoma indicum* species group has received less attention than its congeners since none of these species ultimately infect humans in the final stage of their life cycle [4–6]. However, it remains responsible for severe outbreaks of cattle schistosomiasis and human cercarial dermatitis in India and Southeast Asia [2, 7–12]. *Schistosoma indicum*, *S. nasale*, *S. spindale* and a new lineage recently discovered, *Schistosoma* sp. are the four species constituting this group of mammalian parasites endemic to Asia [5, 6]. All four species use the freshwater bulinine planorbid snail *Indoplanorbis exustus* (Deshayes, 1864) as sole intermediate host during their larval stage. Lymnaeid snail species such as *Lymnaea luteola* were sometimes reported to be infected by species belonging to the *S. indicum* species group, however, both experimental infections and field observations showed that only *I. exustus* positively led to successful infection and larval development [10, 13–15]. In addition to the *S. indicum* group, several other digenean trematode species from the families Sanguinicolidae, Strigeidae, Echinostomatidae, Xiphidiocercariae and Spirorchidae also infect *I. exustus*, although it is not the sole intermediate host [6, 16, 17]. Species of these families cause various severe intestinal, cardiovascular or blood illnesses in animals and humans [18–20]. Given the strict dependence of the *S. indicum* species group on *I. exustus* and the role of this snail in the transmission of several other medically and veterinary important parasites, there is an increased interest in understanding the geographical distribution patterns and evolutionary history of this snail species.

Described for the first time in India, *I. exustus* is widely distributed in Southern Asia, ranging from Iran to China eastwards including India and from the southeastern Himalayas to Southeast Asia southwards (see map range on IUCN [21]). These two regions, grouped into the Indo-Malaya ecozone, record a complex history of geological, tectonic and climatic events that generated striking biogeographical boundaries and decisively shaped the present distribution patterns of living species [22–29].

Liu et al. [30] first investigated the phylogeographical patterns of *I. exustus* in Asia based on thirteen specimens from ten countries and two mitochondrial markers. Using a molecular clock approach they proposed a hypothetical scheme of the spatial and temporal evolution of *I. exustus* in Asia. According to this study, ‘proto-*Indoplanorbis*’ may have originated in northeast Indian (Assam region) and then colonised the northern Indian subcontinent. The authors assumed that the intraspecific divergence started about 7 million years ago (Mya) following the accelerated Himalayan uplift and the establishment of the monsoon climate, resulting in the colonisation of South India. Periodical lowering of the sea level due to the Plio-Pleistocene glaciation oscillations was supposed to have facilitated subsequent dispersal events in Southeast Asia [30].

This study also revealed an unexpectedly high genetic diversity and divergent mitochondrial clades, suggesting the potential existence of more than one species [30]. Another phylogenetic study based on an extended sampling in Nepal went beyond this assumption. Indeed, the analysis revealed four highly divergent mitochondrial clades within this nominal species that seems morphologically homogeneous, at least based on shell morphology. The four clades appear to co-occur in the two investigated localities in Nepal, strongly suggesting that *I. exustus* could actually represent a complex of cryptic species [6]. Because of the host-parasite specificity, the possibility of a cryptic species complex could potentially modify the current patterns of host-parasite associations and thus provide meaningful insights into the spread of each trematode species of the *S. indicum* group. Therefore, a resolution of the taxonomic controversy of *I. exustus* becomes essential.

Liu et al. [30] provided a first step towards the understanding of the species’ phylogeography and genetic diversity in the Indo-Malaya region, while Devkota et al. [6] added a substantial sampling from Nepal. In the present study, we use a larger and more comprehensive sampling throughout Indo-Malaya, including specimens from India and Indonesia, broad geographical areas for which genetic data are still scarce. More specifically, we built a dataset of 97 sequences of a partial mitochondrial gene fragment (cytochrome *c* oxidase), compiling sequences from previous studies with 53 sequences new to this study. In particular, sequences were added for South India and for Indonesia, extending coverage for this region from a single sample in West Java to West Papua through Sulawesi and North Moluccas.

Our objectives are (i) to test whether genetic analyses based on an extended sampling support a complex of cryptic species, (ii) to examine the historical patterns of diversification over its entire distribution range, and (iii) to test whether the fragmented nature of the Indo-Australian Archipelago (IAA) and particularly the presence of the Wallace Line, a recognized zoogeographical barrier within this archipelago, could affect the genetic structure and diversity of this species in this newly investigated area. Finally, we will discuss these results considering the inherent concerns of parasitology.

## Methods

### Sampling, DNA extraction, amplification and sequencing

Specimens from Southeast Asia and the Indian subcontinent were collected in the field or were provided by other research institutions and museums. The sampling comprised 31 specimens from Indonesia, 13 specimens from India, 4 specimens from Myanmar, 3 specimens from Laos and 2 from Thailand. The specimens were manually collected using a sieve in stable pools, ponds, rice fields, marshes and slow running waters as well as ephemeral puddles. The specimens were stored in 80% ethanol. Total DNA was extracted from the snail foot muscle with the Qiagen DNeasy® Blood and Tissue Kit (Hilden, Germany) following the manufacturers’ instructions. A 655 bp long fragment of the mtDNA-encoded cytochrome *c* oxidase subunit 1 (*cox*1) gene was amplified for 53 specimens with the primer pair used in Wilke et al. [31].

Amplifications were done using 20 μl reaction containing 0.5–1 μl template DNA, a final concentration of 0.1 U *Taq* Polymerase (New England BioLabs®, Ipswich, England), 1× ThermoPol® Reaction Buffer (New England BioLabs®), 0.175 mM of each dNTP, 0.175 mM MgCl_2_, 5 mM TMAC, 0.6 mg/ml BSA and 0.7 μM of each primer. Cycling parameters included one pre-denaturation step of 2.5 min at 94 °C, followed by 40 cycles each consisting of 30 s at 90 °C, 1 min starting at 60 °C and decreasing of 0.3 °C every cycle (touchdown method) and 1 min at 72 °C, followed by a 5 min final extension at 72 °C. Specificity and yield of amplifications were checked via agarose gel electrophoresis. Sequencing was performed by LGC Limited Genomics Berlin in both directions (GenBank accession numbers: KY024420–KY024472, Additional file 1: Table S1).

### Phylogenetic analyses

Sequence editing was manually carried out with the software MEGA 6.0 [32]. The dataset was complemented with 13 sequences published by Liu et al. [30], 30 sequences published by Devkota et al. [6] and one sequence published by Albrecht et al. [33] resulting in a total of 97 specimens. The alignment was performed by eye and then trimmed to 618 bp to match the short sequences from Liu et al. [30]. *Bulinus tropicus* which, as *I. exustus*, belongs to the Bulininae tribe and *Burnupia caffra*, another planorbid species, were used as outgroups. The *cox*1 dataset was reduced to unique haplotypes and tested for nucleotide substitution saturation using the implemented test by Xia & Xie [34] in DAMBE 5.3 [35]. No significant saturation was detected, even under the very conservative assumption of an extremely asymmetrical tree [36].

Phylogenetic analyses were performed using Bayesian inference (BI) and maximum likelihood (ML) methods. ML analyses were performed using PhyML 3.0 with default settings [37]. The best substitution model for this dataset was HKY + Γ according to jModelTest2 [38]. Statistical support was obtained by calculating 500 bootstrap replicates.

The BI phylogeny was inferred with MrBayes 3.2.1 [39]. We tested the *cox*1 dataset for partitions using PartitionFinder2 (model of evolution: MrBayes; model of selection: AICc; scheme search: all) [37, 40]. The best partition scheme suggested the following subpartitions and substitution models: codon position 1: GTR + I + Γ, codon position 2: GTR + I + Γ, codon position 3: HKY + Γ. Two independent Markov Chain Monte Carlo (MCMC) searches were run for 20 million generations, sampled every 1000 generations, each with four chains and a temperature of 0.1 and a stop value of 0.01 used as a convergence diagnostics. Ten percent of the samples were discarded as burnin. Convergence of the two independent runs was examined *a posteriori* by plotting the generated log files in Tracer 1.5 [41]. Effective sample size (ESS) of at least 200 indicates adequate sampling of posteriors distributions.

### Genetic diversity

Uncorrected p-distances were calculated with MEGA 6.0 and summarised within and among the major mitochondrial clades inferred from the phylogenetic analyses.

Genetic diversity within clades was estimated with the following indices using DnaSP 5.10 [42]: numbers of haplotypes, haplotype diversity (h), numbers of polymorphic sites (S) and nucleotide diversities (π).

### Estimation of divergence times

We estimated divergence times through the BEAST 1.8.4 package [43] using a constant size coalescent tree prior. The coalescent approach is dependent on the effective population size, which is estimated by the program using the total number of taxa and frequency for each haplotype. For this reason, the dataset included all the individuals initially sequenced as well as all the formerly published sequences, even if they shared the same haplotype. There is theoretically no need for specifying an outgroup when running BEAST analyses. However, we included *Bulinus tropicus* to get a rough estimation of the divergence time between the two sister-species.

The dataset was partitioned by codon position as suggested by PartitionFinder2 (see above for the settings): codon position 1: TrN + Γ, codon position 2: HKY + I, codon position 3: HKY + Γ.

We tested three clock models: a strict clock, an uncorrelated lognormal relaxed (UCLN) clock with a continuous quantile parameterization [44] and a random local (RLC) clock. As no internal clock rate or fossils are described yet for this particular species, we used a *cox*1-specific external clock rate established for aquatic Protostomia by Wilke et al. [45]. In this study, the authors estimated trait-specific clock rates for commonly used substitution models. The estimated clock rates were similar among these models, however, they differed considerably when using gamma distribution and invariable site parameters The best-fit substitution model for the whole *cox*1 gene fragment is HKY + Γ according to the AICc and BIC obtained by jModelTest 2.1.5 [38]. Because clock rates were only estimated for the models HKY and HKY + I + Γ in the above-mentioned source study [45], we used the mean value and confidence intervals of the two molecular clock rates (rate = 1.405% of substitutions/site/million years, fixed prior, stdv = 0.335% of substitutions/site/million years, fixed prior). Analyses were run three times independently with a chain of 75 million generations sampled every 2500 generations. We originally started with 20 million generations and progressively increased this number until the runs reached convergence and ESS values > 200.

The marginal likelihoods for each run were estimated using the path sampling (PS)/stepping stone sampling (SS) methods implemented in BEAUti 1.8.4 [43] to compare the molecular clock models. The mean values of the SS and PS logarithm marginal likelihoods from the three runs were calculated. Based on the marginal likelihood estimates (MLE), the RLC model was favoured over the UCLN model and the strict-clock model for both analyses (PS and SS) (Additional file 2: Table S2).

We examined the performance *a posteriori* for the molecular clock analysis with the program TRACER 1.6 [41] and accepted results when ESS values were > 200. The first 10% of the sampled trees were discarded as burn-in and the Maximum Clade Credibility tree (MCC tree) was obtained from the resulting trees using TreeAnnotator (BEAST 1.8.4 package; [43]). The MCC tree with the divergence times was visualised in FigTree 1.4 [46].

### Phylogeographical analysis

Relationships among the haplotypes were identified by constructing a minimum spanning network with a connection limit of 95% using TCS 1.21 [47]. The geographical occurrence was assigned to each haplotype.

### Population structure and demographic inference within the Indo-Australian Archipelago (IAA)

The analysis of molecular variation (AMOVA) was conducted to determine the genetic variation and population structure of *I. exustus* within the IAA in Arlequin 3.5 [48]. The dataset included 33 individuals that were grouped by islands. We excluded the Philippines and the Malaysian peninsula since the sampling for these areas included only a single specimen each. The amount of genetic variation and structure within the IAA was investigated at different geographical levels: (i) within and (ii) among islands and (iii) between two biogeographical groups separated by the Wallace Line (western part: Java, Borneo and eastern part: Sulawesi, Halmahera and West Papua), in order to test whether this zoogeographical barrier could have affected the genetic structure of *I. exustus*. The AMOVA was completed with 10,100 permutations.

We performed neutrality tests using the program Arlequin 3.5 [48] to test the hypothesis of population expansion within the IAA. We employed the traditional Fu’s Fs and Tajima’s D neutrality tests as an assessment of possible population expansion. Additionally, we used DnaSP 5.0 [42] to calculate the Fu & Li’s D* and Fu & Li’s F*, two tests that were shown to be powerful to detect sudden demographic changes [49]. Under the assumption of neutrality, an expanding population results in significant large negative values for all indices.

Population expansion events were determined by computing mismatch distributions under the sudden demographic and spatial expansion models using Arlequin 3.5 [48]. The number of bootstrap replicates was set to 1000. Unimodal distribution of pairwise differences is usually the sign of a recent population growth. The goodness of fit of the observed to the expected pairwise differences predicted by the models was tested using the sum of square deviation (SSD) and Harpending’s raggedness index (HI). Non-significant values for these two tests (*P* > 0.05) indicate that the model cannot be rejected, i.e. a good fit of the data to the model.

Given that any of those expansion models are accepted, the putative time of population expansion can be estimated from the statistic tau (τ) (expressed in units of mutational time) calculated in Arlequin 3.5 [48] using the following function (see [50]):

$$ T\left(\uptau, \upmu \right)=\frac{\uptau}{2 mT\upmu} $$

where *mT* is the number of base pairs in the dataset (here 618 bp) and μ is the mutation rate per nucleotide (1.22% My^-1^ as defined by Wilke et al. [45] under the most simple Jukes-Cantor substitution model). Given that tau’s upper and lower bounds were too divergent from the mean value, ΔT was instead calculated by using the standard deviation of the mutation rate provided in [43] (±0.27% My^-1^).

## Results

### Phylogenetic relationships

Bayesian inference and maximum likelihood methods revealed congruent topologies including five well-supported mitochondrial clades. Relationships between the clades were mostly well supported (Additional file 3: Figure S1).

**Fig. 1:**
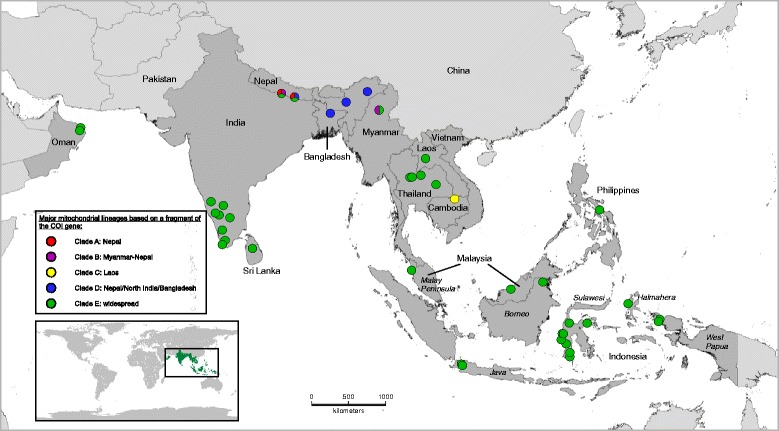
Distribution of the major mitochondrial clades for *Indoplanorbis exustus*. Five main phylogenetic clades were identified. Colours refer to the main mitochondrial clades based on a *cox*1 gene fragment. Pie charts indicate co-occurrence of distinct mitochondrial clades at the same locality (see Fig. 2 for phylogenetic relationships)

Based on this dataset, the geographical distribution of clades shows major differences, one clade exhibiting a much larger distribution range than the others in the studied area (Fig. 1). The basal clade A consists exclusively of specimens from Nepal. Clade B comprises specimens from Nepal and Northern Myanmar (Lake Indawgyi). The strongly supported sister clades C and D are geographically restricted to southern Laos and the North Indian subcontinent (Nepal, North India, Bangladesh), respectively. Finally, clade E comprises a large number of specimens widely distributed in the Indo-Malayan region ranging from the Indian subcontinent (Sri Lanka, India, Nepal, Bangladesh) to Southeast Asia (Myanmar, Thailand, Malaysia, Philippines and the Indonesian archipelago).

Several clades co-occurred in different geographical areas: four of the five clades occur in Nepal, while representatives of the clades B and E co-occurred in North Myanmar (Fig. 1).

### Genetic diversity

The dataset revealed 36 unique haplotypes among the 97 specimens. High levels of haplotype and nucleotide diversities (h: 0.889 ± 0.026; π: 0.067 ± 0.005) were observed among all specimens (Table 1).

**Table 1:** Estimates of genetic diversity for major *cox*1 haplotype clades of *Indoplanorbis* in Asia

Clade	Sample size	Haplotypes	Polymorphic sites (S)	Haplotype diversity (h)	Nucleotide diversity (per site) (π)
A	9	6	13	0.889 ± 0.091	0.006 ± 0.002
B	18	6	12	0.745 ± 0.079	0.005 ± 0.001
C	3	3	8	1.000 ± 0.272	0.008 ± 0.003
D	6	4	63	0.800 ± 0.172	0.058 ± 0.011
E	61	21	47	0.747 ± 0.058	0.010 ± 0.001
Total	97	36	153	0.889 ± 0.026	0.067 ± 0.005

The uncorrected p-distances revealed high levels of genetic divergence among clades. The overall mean p-distance is 8.6% (Table 2). The genetic distance between clades is at least 7.3% (except for the sister clades C and D). The maximal genetic distance is 13.9% between the clades A and D, both occurring in Nepal. Genetic distances were 10 times lower within than between the clades.

**Table 2:** Uncorrected p-distance ranges for *cox*1 gene sequence (%) among and within (bold) mitochondrial gene clades of *Indoplanorbis exustus*

	Clade A	Clade B	Clade C	Clade D	Clade E
Clade A (Nepal)	**0.0–1.9**	–			
Clade B (Nepal-Myanmar)	11.8–12.8	**0.0–1.8**	–		
Clade C (Laos)	12.3–13.1	9.4–10.7	**0.0–1.9**	–	
Clade D (Nepal-North India)	12.8–13.9	10.7–11.2	4.4–5.2	**0.2–1.3**	–
Clade E (widespread)	11.8–13.3	10.5–12.1	7.6–10.0	7.3–9.2	**0.0–3.9**

High haplotype diversity was observed for each mitochondrial clade, ranging from 0.745 ± 0.079 for clade B to 1.000 ± 0.272 for clade C (Table 1). The nucleotide diversity was low for all clades (≤ 0.01) except for the North Indian subcontinent clade (D), which was more than five times higher than in the other clades.

### Estimation of divergence times

**Fig. 2:**
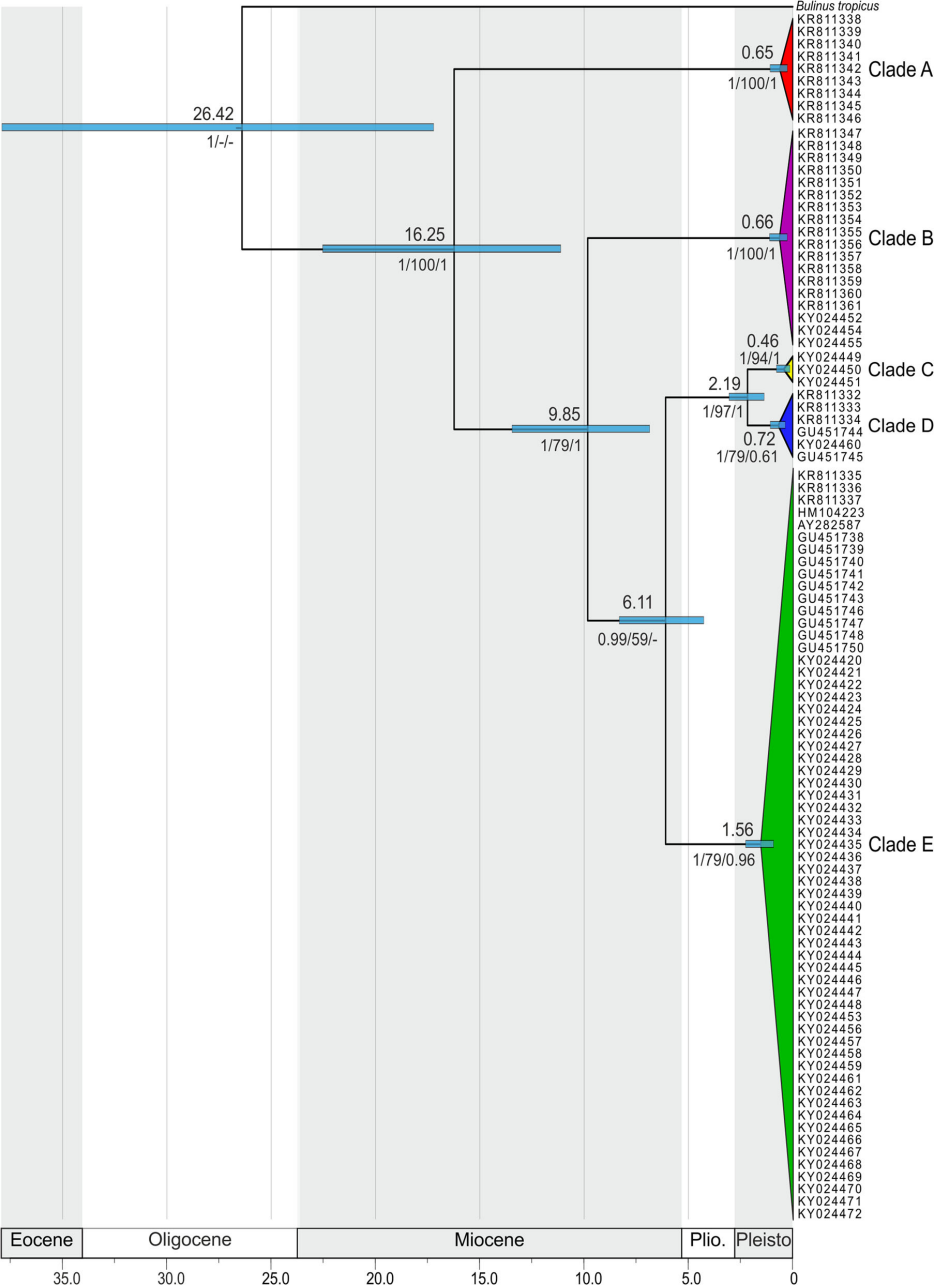
Strict-clock MCC tree based on *cox*1 for *Indoplanorbis*. Estimated mean ages of selected evolutionary events are shown on top of the nodes in million years including error bars (*light blue*). Numbers below nodes indicate BEAST posterior probabilities, ML bootstrap values and MrBayes posterior probabilities, respectively. *Abbreviations*: *Plio* Pliocene, *Pleisto* Pleistocene

The MCC tree obtained by coalescent inference from the BEAST analysis revealed the very same five highly supported phylogenetic clades inferred by the ML and BI approaches (Fig. 2). The topology was slightly different from those inferred by the ML and BI analyses. While the position of clade B was ambiguous in the ML and BI phylogenetic trees (Additional file 3: Figure S1), the BEAST analysis suggests clade B as being the sister clade to the clades C, D and E.

According to the Bayesian divergence time estimates, the separation of Asian *Indoplanorbis* from its African sister species group, *Bulinus* spp., occurred in the Late Eocene-Early Miocene (17.24–37.93 Mya, 95% HPD, highest posterior density, interval). From the Early Miocene to the Plio-Pleistocene transition, four major splits occurred at 16.25 Mya (11.15–22.55 Mya), 9.85 Mya (6.88–13.48 Mya), 6.11 Mya (4.29–8.34 Mya) and 2.19 Mya (1.40–3.07 Mya), resulting in the five recent mitochondrial clades. Intra-clade diversification occurred rather simultaneously, roughly within the Middle Pleistocene.

### Phylogeographical patterns

**Fig. 3:**
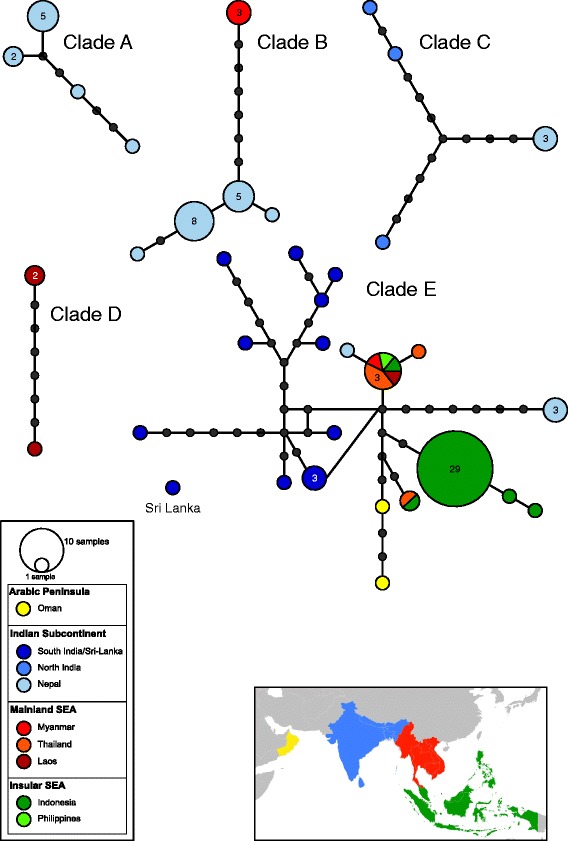
Phylogeography of *Indoplanorbis exustus*. Minimum-spanning parsimony network showing the hierarchical relationships between 36 unique *cox*1 haplotypes with a 95% connection limit. Haplotypes are coloured according to their geographical occurrence. A colour gradient is used to group countries belonging to the same biogeographical region (Arabic Peninsula in *yellow*, Indian subcontinent in *blue*, mainland Southeast Asia (SEA) in *red* and insular SEA in *green*; see the map in Fig. 1). *Circles* are sized proportionally to the frequency of occurrence, ranging from 1 to 29 (the number of individuals sharing the same haplotype is indicated in the circles). Each line between points represents a single mutational step

The TCS analysis revealed six disconnected haplotype groups consisting of five independent haplotype networks and one single haplotype belonging to the unique specimen from Sri Lanka (Fig. 3). This latter haplotype would be connected to the most complex haplotype network under a parsimony connection limit of 90% (13 mutational steps from the closest haplotype of a specimen from India), resulting in five haplotype networks consistent with the five phylogenetic clades.

According to their geographical occurrence, the haplotypes were assigned to four major biogeographical regions that have distinct biota assemblages and peculiar tectonic, geological and climatic histories: the Arabic Peninsula (Oman), the Indian subcontinent (Sri Lanka, India, Bangladesh, Nepal), continental Southeast Asia (Indochina: Myanmar, Laos, Vietnam, Thailand, Cambodia) and insular Southeast Asia also known as the Indo-Australian Archipelago (IAA) (Malay Peninsula, Indonesia, Philippines) (Fig. 3). Although one network included haplotypes exclusively found in Nepal, patterns of genetic structure are not clearly reflecting the geographical/regional patterns. The most complex network corresponding to the clade E included haplotypes widely distributed across the four biogeographical regions.

The Indian subcontinent comprised the greatest haplotype diversity with 25 haplotypes assigned to five independent networks. Four of these five networks included haplotypes from Nepal (light blue), revealing a high level of genetic divergence occurring in relatively restricted areas and sometimes in sympatry. The group of continental Southeast Asia comprised eight haplotypes assigned to three independent networks (red colour code). Finally, despite the large geographical area and the comparatively high number of specimens sequenced (34 individuals from Borneo to New Guinea), the IAA exhibited the lowest number of haplotypes (six) all belonging to one single network. Within this network, 29 individuals widely distributed across the Indonesian archipelago share the same haplotype. The haplotypes of the other individuals found on the IAA were only one to four mutational steps divergent from the main haplotype (see green colour code on the Fig. 3).

Nucleotide and haplotype diversities were much higher on the continent (Indian subcontinent and mainland Southeast Asia) than within the IAA, although the number of specimens on the archipelago represented about one-third of the total dataset (Additional file 4: Table S3). The Indian subcontinent exhibited the highest nucleotide and haplotype diversities (Additional file 4: Table S3). The genetic variation within the Arabic Peninsula was not investigated since this region was only represented by two individuals.

### Geographical structure and demographic inference of the IAA subclade

According to the AMOVA, the greatest amount of variation was within the islands of the IAA (78.53%), while the amount of variation between biogeographical units and among islands within the biogeographical units was ~ 10% each. This indicates an absence of differentiation between the insular populations, as well as between the two biogeographical units separated by the Wallace Line. None of the three fixation indices was statistically significant, rejecting an insular- or biogeographical-dependent population genetic structure (Table 3).

**Table 3:** Analysis of molecular variance (AMOVA) within the Indo-Australian Archipelago. The regions at the eastern and western sides of the Wallace line constitute the compared biogeographical units

Source of variation	*df*	Sum of squares	Variance components	Percentage of variation	Fixation Index (*P*-value)
Among biogeographical units	1	0.658	0.024 Va	10.6	F_CT_ = 0.106 (0.203)
Among islands within biogeographical units	3	1.003	0.024 Vb	10.8	F_SC_ = 0.121 (0.219)
Within islands	28	5.067	0.180 Vc	78.5	F_ST_ = 0.214 (0.080)
Total	32	6.727	0.230		

*Abbreviation*: *df* degrees of freedom

This subclade exhibited low haplotype and very low nucleotide diversities. The Tajima’s D, Fu & Li’s D* and Fu & Li’s F* values were all negative and statistically significant, supporting the hypothesis of a sudden population expansion (Table 4).

**Table 4:** Estimates of genetic diversity and tests of neutrality for the population of the Indo-Australian Archipelago

Estimates of genetic diversity					Neutrality tests			
Sample size	H	S	h	π	Fu’s Fs	Tajima’s D	Fu & Li’D*	Fu & LiF*
33	4	6	0.176 ± 0.088	> 0.001 ± > 0.001	-1.530	-1.995**	-2.751*	-2.942*

*P < 0.05; **P < 0.01

*Abbreviations*: *H* number of haplotypes, *S* number of polymorphic sites, *h* haplotype diversity, *π* nucleotide diversity

**Fig. 4:**
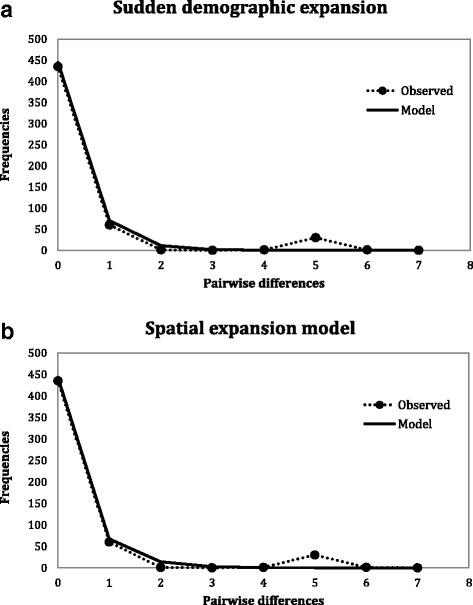
Observed and expected mismatch distributions for the total population of the IAA. The observed distributions (*black points* and *dotted line*) are compared for their goodness-of-fit to a Poisson distribution under a model of sudden population expansion (**a**) and a model of spatial expansion (**b**)

The mismatch analysis showed an unragged bimodal profile with a pronounced narrow base, similar to the expected distribution profiles under both sudden demographic and spatial expansion models (Fig. 4). For each model, both the SSD and the HI statistical tests were not significant for each model and thus the sudden demographic and spatial expansion cannot be rejected for this subclade (Table 5). The time of demographic expansion (T_demographic_) was estimated about 198,950 years (ΔT_demographic_: 162,898–255,493 years); the time of spatial expansion (T_spatial_) was estimated about 389,079 years (ΔT_spatial_: 318,575–499,659 years) (Table 5).

**Table 5:** Estimated parameters of the sudden demographic and spatial expansion models for the population of the Indo-Australian Archipelago based on the distribution of pairwise nucleotide differences

Model	SSD	P (SSD)	HI	P (HI)	τ	T [y]	ΔT [y]
Sudden demographic expansion	0.021	0.160	0.431	0.620	3.000	198,950	162,898–255,493
Spatial expansion	0.007	0.490	0.431	0.720	5.867	389,079	318,575–499,659

*Abbreviations*: *SSD* sum of squared deviation, *HI* Harpending’s raggedness index, *τ* estimator of the mutational time since the occurrence of the population expansion in units of mutational time, *T* time since expansion in years

## Discussion

### Taxonomic status of *Indoplanorbis exustus*

As previously proposed in other phylogenetic studies [6, 30], our results based on a more comprehensive sampling throughout the Indo-Malaya region strongly suggest that *I. exustus* represents a cryptic species complex. First, the phylogenetic analyses revealed five highly divergent clades, regardless of which approach has been applied. The new specimens examined clustered within three of the four Nepalese clades originally identified by Devkota et al. [6], while a fifth clade comprising specimens from one locality in Laos was uncovered (Fig. 2 and Additional file 3: Figure S1). This pattern is also supported by the network analysis that revealed five unconnected clade-specific haplotype networks, except for the haplotype from Sri Lanka that clustered within clade E (Figs. 2 and 3). The network analysis is thought to accurately distinguish closely related species by clustering individuals belonging to the same species based on the 95% level [51]. Furthermore, initial diversification between clades began early in the Early-Middle Miocene (11.15–22.55 Mya), while intra-clade diversification occurred more recently during the Pleistocene, suggesting a long-term disruption of gene flow based on the mitochondrial marker used (Fig. 2). Additionally, high genetic distances among the clades ranging from 4.4 to 13.9% (mean distance of 8.6%) confirm a pronounced isolation (Table 2). Similar or even lower values were reported for species pairs of the genus *Bulinus*, the sister-group of *Indoplanorbis*, namely 2.9% within the *B. trunctus*/*tropicus* species complex and 5.2% between *B. nyassanus* and *B. succinoides* [52]. Finally, the fact that some clades occur sympatrically in Nepal and Myanmar strengthen the argument of a disrupted gene flow (Figs. 1 and 3).

Considering such genetic divergence, one might expect to identify divergent characters among the clades. However, the examination of the shell as well as the comparison of the male copulatory organ, characters that are commonly used to distinguish species of this gastropod family [53], did not provide conclusive diagnostics (data not shown).

Based on the definition proposed by Bickford et al. [54], the existence of these genetically highly divergent and morphologically indistinguishable clades within a presumed nominal species strongly support the assumption that *Indoplanorbis exustus* may, in fact, be a complex of five cryptic species. An increasing number of phylogeographical studies focusing on “widespread” species in Southeast Asia and the Himalayas revealed unexpected cryptic diversity, alternatively attributed to the past climate changes and/or the complex geological history of the region [55–60]. It is, therefore, important to identify the factors that have triggered diversification in the *Indoplanorbis* species complex.

### Historical biogeography of the *Indoplanorbis* species complex

The estimates of divergence times combined with the analysis of the geographical distribution of the clades and haplotypes allow us to infer origin and diversification patterns of the *Indoplanorbis* species complex in Asia over space and time.

Two biogeographical scenarios with different chronologies have been formerly proposed to explain the divergence between *Indoplanorbis* and its sister species-group *Bulinus* spp.: (i) a Gondwanan origin implying a vicariant event when the Indian terrane broke off from Africa about 125 Mya ago and drifted northward to collide with the Asian continent about 50 Mya ago, and (ii) a Miocene Sinai-Levant dispersal out of Africa into Asia following the closure of the eastern end of the Tethys Sea [30, 61, 62]. According to our molecular dating, *Indoplanorbis* initially diverged from its African sister group *Bulinus* spp. (used as outgroup) in the Late Eocene-Early Miocene, between 17 Mya and 38 Mya (Fig. 2). Even when considering the lowest bound of the age interval, the estimated timeframe post-dates the first mentioned vicariant event and rather coincides with a Miocene dispersal event along the Middle East land connection, which was already assumed by Morgan et al. [62]. The fact that none of the individuals found in South India belongs to the basal clade reinforces the assumption that *Indoplanorbis* did not originate in India and thus again argues against a Gondwanan rafting hypothesis.

The main genetic diversity is found in the lowlands of Nepal, where all but one clade occur in the relatively restricted area (Figs. 1, 2 and 3). This strongly suggests that proto-*Indoplanorbis* might have originated in the humid subtropical plains of Nepal or in unstudied southern adjacent areas such as northern India as suggested by Liu et al. [30]. It is unlikely that this tropical species group originated in a more northern region where the highland water temperatures are drastically colder and thus inhibit the completion of the life cycle [63]. However, more extensive sampling in this region as well as in southwestern Asian countries (e.g. Pakistan, Afghanistan, Iran) is needed for a final conclusion as to this pattern.

The five phylogenetic clades within *Indoplanorbis* arose successively from the Early Miocene to the Plio-Pleistocene transition and further diversified during the Pleistocene (Fig. 2). While Liu et al. [30] suggested an initial divergence in the Late Miocene about 7 Mya and lasting until the Middle Pleistocene, the present analysis suggests an earlier time of diversification. This can be explained by the fact that all the clades considered by Liu et al. for interpreting the phylogeographical patterns cluster within the widespread clade E in this study, except for the North Indian clade that is placed within the clade (D).

The Mid-Late Miocene was a period of tectonic and climatic instability in Southern and Southeast Asia. Following the rapid uplift of the Qinghai-Tibetan Plateau (QTP), the East Asian monsoon initiated in the Late Oligocene [64, 65] and may subsequently have periodically intensified three times about 13–15 Mya, 8 Mya and 3 Mya [65–67]. These periods of intensification have been associated to the Himalayan orogeny, the retreat of the Paratethys Sea from Central Asia as well as to the initiation of the major onset of the Northern hemisphere glaciation during the late Pliocene [68–70].

According to the divergence time estimates and their credibility intervals, the three first major diversification events dated at about 16.25 (11.15–22.55) Mya, 9.85 (6.88–13.48) Mya and 6.11 (4.29–8.34) Mya and thus predate or coincide with the time frames of the respective intensification periods of the East Asian monsoon. The considerable aridification and intensification of the Asian monsoon that occurred 8 Mya has been proposed to have triggered diversification in *Indoplanorbis* [30]. It has also been linked to divergence events in other freshwater and terrestrial taxa as suggested for the Triculinae [71], another family of freshwater gastropods intermediate host for distinct *Schistosoma* species affecting humans, and for species groups of freshwater fishes [72], amphibians [55] and birds [73].

The cladogenetic event between clades C and D is dated at about 2.19 Mya and could have been promoted by the Plio-Pleistocene climatic oscillations resulting from the repeated glacial cycles, which followed the formation of the Northern Hemisphere ice sheets 2.7 Mya [69]. Interglacial dispersal events may have contributed to the range expansion of the clades while population isolation in humid refugia zones during the glacial periods may have fostered the intra-clade diversification in the Middle-Late Pleistocene and even possibly the divergence between the two clades C and D [26].

Contrary to our expectations, no correlation was evident between the current geographical distribution of the clades and the major biogeographical regions in this area (Indian subcontinent, Southeast Asia) (Fig. 3). However, distribution patterns could be related to either the course of the main rivers such as the Brahmaputra for clade D and the Mekong for clade C in Laos or humid refugia zones such as the ancient lake Indawgyi in Myanmar (Clade B) [26, 74, 75]. The evolving river systems resulting from the Himalayan orogeny were suggested as the main dispersal route for the Triculinae, while ancient lakes of Myanmar and Yunnan Province in China may have served as refugia during harsher glacial climatic conditions during the Pleistocene [76, 77]. The occurrence of similar biogeographical patterns reinforces the plausibility of this assumption. A more comprehensive sampling for each clade will undoubtedly provide a more detailed picture and will help to determine the factors implicated in the current distribution of the clades.

In contrast to the other clades, clade E shows a very different geographical pattern with a widespread distribution range over the entire Indo-Malayan region and even including specimens from the Arabic Peninsula. Such discrepancy compared to the other clades in the distribution pattern may reflect that this clade developed distinct intrinsic properties such as a greater dispersal capability and the ability to inhabit various types of habitats.

Interestingly, the specimens sampled in the IAA all belong to the ecologically variable clade E (Figs. 1 and 3). The question arises of how this clade reached and dispersed throughout the archipelago, a region that is for the first time systematically investigated.

### Recent range expansion in the Indo-Australian Archipelago (IAA)

A large and representative sampling coverage across the IAA allowed exploring the genetic structure of clade E, the only clade of the *Indoplanorbis* species group present in this less studied geographical area.

Because of their restricted size and their isolated and discontinuous nature, islands are thought to hamper dispersal for terrestrial and freshwater organisms. However, several studies have shown that dispersal across the IAA has occurred more often than previously expected [24, 27, 28]. The combination of inter-island reduced gene flow and intra-island genetic drift often results in genetically differentiated populations from the mainland and to a lesser extent, among the islands [78].

However, contrary to our expectations, the insular populations of clade E, for which relatively high levels of genetic diversity were detected on the mainland, did not exhibit any genetic structure on the archipelago (Fig. 3, Tables 3 and 4). Indeed, the AMOVA did not reveal population genetic structure neither among islands nor between the two sides of the widely recognised zoogeographical Wallace’s Line, which strongly suggests a recent colonisation independent of natural biogeographical constraints (Table 3). The negative and significant neutrality tests as well as the acceptance of the two mismatch distribution models support a sudden demographic and spatial expansion (Fig. 4 and Table 4). This may have occurred in the Middle-Late Pleistocene, the spatial expansion predating the demographic expansion (about 318,575–499,659 years ago and 162,898–255,493 years ago, respectively). These results suggest a recent colonisation by natural dispersal. In almost two centuries of malacological exploration of the IAA, *Indoplanorbis* has only been recorded in 1897 in Sumatra [79], the botanical Garden of Bogor in Java in 1946 [80] (rediscovered in 2005 [30]) and Sulawesi in 1956 [81]. To our knowledge, no more recent records have been published for other parts of the archipelago up to now. This, however, rather indicates a modern stepping-stone dispersal event from the northeast to the south-west of the IAA most likely fostered by anthropogenic activities. *Indoplanorbis* was introduced and sometimes well established in several regions far from its native distribution range (in Ivory Coast [82], in Benin [83], in Nigeria [84] and in French West Indies, Guadeloupe [85]). Similar patterns of recent (possibly human-mediated) range expansion with extremely low genetic diversity within the IAA contrasted to relatively diverse and structured patterns on the mainland were already observed in frogs and civet cats [56, 86].

Considering all these findings, the *Indoplanorbis* clade in the IAA could represent a potentially invasive species and emerge as an ecological threat to the native species in this biodiversity hotspot. Furthermore, because of its role in the spread of *Schistosoma* parasites, it becomes of real medical and veterinary concern.

### Concerns for parasitology

*Indoplanorbis* is considered as the sole intermediate host responsible for the *S. indicum* species group and is involved in the transmission of several other trematode parasites [6, 13]. Even though this study focuses exclusively on the *Indoplanorbis* species group, it seems evident that our findings may also have relevance for achieving a more comprehensive understanding of the relationship of *Indoplanorbis* and its component clades with digenetic trematodes of veterinary or medical significance.

The origin of the *S. indicum* group has been frequently debated, but a recent study using DNA sequencing, comparative molecular genomics and karyotyping strongly supports a colonisation of Asia from Africa across the Sinai-Levant dispersal tract triggered by the mass movement of large mammals [87]. This geographical scenario, similar to the one suggested for *Indoplanorbis*, is supposed to have occurred between the Pliocene and the Pleistocene (2–3 Mya). Since the first presumed passage of its intermediate host occurred in the Late Eocene-Early Miocene, it would provide a sufficient timeframe in order to disperse to Asia and become established. The diversification of the *S. indicum* group in South and Southeast Asia is not well established yet. However, the emergence of the different species forming the group is thought to have occurred lately during the Late Pleistocene [5], a period coinciding with intra-clade diversification processes in *Indoplanorbis*. Parasite dispersal and geographical diversification are strongly related to host dispersal ability and degree of connectivity among host populations [88]. Therefore, a more comprehensive phylogeographical study focusing on both the host and the parasite as well as a better characterization of the ecological preferences and life history traits for each *Indoplanorbis* clade is needed for monitoring the spread of *Schistosoma* and the development of prevention and mitigation plans.

Given that *Indoplanorbis* might represent a species complex, it also becomes necessary to reconsider the host-parasite relationships. Devkota et al. [6], who first proposed the existence of a snail-host complex, could not identify any specific host lineage-parasite species relationships based on their parasitological screening. This suggests that *S. indicum* may be able to switch the hosts within the *Indoplanorbis* species complex.

Based on the results from Devkota et al. [6], the individuals found in Nepal and belonging to the widespread clade E in our study did not shed any species of the *S. indicum* group. However, an elaborate parasitological screening of several host individuals belonging to this clade and from other regions is necessary to draw conclusions about the potential role of this clade in the transmission of this group of parasites. Besides, strigeids, xiphidocercariae and sanguinicolids parasites were reported to be hosted by individuals from this clade, which confirms the importance of an enlarged parasitological survey, despite the absence of host specificity for *Indoplanorbis*. The reduced genetic diversity of the insular population is also worrying from a parasitological viewpoint since host populations exhibiting low genetic diversity have been shown to be less resistant to parasite infections and thus contribute significantly to parasite transmission [89]. Given its wide geographical distribution and the recent range expansion in the IAA, it becomes essential given medical and veterinary concerns to survey the spread of the species in order to genotype and to monitor parasite infections.

## Conclusions

Several lines of evidence lead to the conclusion that *Indoplanorbis exustus* represents a complex of five cryptic species. *Indoplanorbis* diverged from its sister species group *Bulinus* about 26 Mya. It is assumed that proto-*Indoplanorbis* dispersed from Africa to South Asia through the Middle East land bridge of the Sinai Levant in the Miocene. The divergence within the species complex occurred between the Early Miocene and the Plio-Pleistocene transition and might have been associated with the monsoonal regime fluctuations. The intra-clade diversification took place simultaneously during the Pleistocene, a period of repeated climatic changes due to the glacial/interglacial oscillations. We witness a recent range expansion into the Indonesian archipelago that is likely human-mediated. The existence of a cryptic species complex, the historical divergence patterns and the distribution of clades as well as the recent range expansion imply important ecological and medical concerns related to parasitology and public health.

In the future, broader sampling including specimens from North India and western South Asia may provide more insights into the biogeographical patterns of this cryptic species complex. Additionally, faster-evolving genetic markers and more specific population genetic analyses on the archipelago could help to estimate and predict the spread of the potentially invasive species. Finally, further morphometric and ecological analyses are needed to disentangle the species complex and to assess the consequences for parasitology. Such efforts should be complemented with parallel biomedical studies.

## Additional files

Additional file 1: Table S1. List of the samples included in this study. For each specimen, the GenBank accession number for the associated *cox*1 sequence, the authors who published the sequences and the collection date, locality and coordinates of the sample are provided. (XLSX 56 kb)
Additional file 2: Table S2. Clock model comparison. (DOCX 19 kb)
Additional file 3: Figure S1. Phylogeny of *Indoplanorbis exustus* estimated by Bayesian inference and maximum likelihood. (PDF 19 kb)
Additional file 4: Table S3. Genetic summary statistics per biogeographical regions for all clades. (XLS 26 kb)

## Abbreviations

AIC: Akaike information criterion
AMOVA: Analysis of molecular variance
BI: Bayesian inference
BIC: Bayesian information criterion
*cox*1: cytochrome *c* oxidase subunit 1 gene
ESS: Effective sample size
HI: Harpending’s raggedness index
HPD: Highest posterior density
IAA: Indo-Australian Archipelago
MCC: Maximum clade credibility
MCMC: Markov chain Monte Carlo
ML: Maximum likelihood
Mya: Million years ago
Pleisto: Pleistocene
Plio: Pliocene
QTP: Qinghai-Tibetan Plateau
SEA: South-east Asia
SSD: Sum of squared deviation
