# Supplementary material for: Recent range expansion of an intermediate host for animal schistosome parasites in the Indo-Australian Archipelago: phylogeography of the freshwater gastropod Indoplanorbis exustus in South and Southeast Asia
Source: Parasit Vectors. 2017 Mar 6;10:126. doi: 10.1186/s13071-017-2043-6 (PMC5339995; doi:10.1186/s13071-017-2043-6)
Supplement: Additional file 2: Table S2. — Clock model comparison. (DOCX 19 kb) [file 13071_2017_2043_MOESM2_ESM.docx]

| **Clock Model** | **Run** | **lmL(SS)** | **mean lmL(SS)** | **BF** | **lmL(PS)** | **mean lmL(PS)** | **BF** |
| --- | --- | --- | --- | --- | --- | --- | --- |
| Strict | run 1 | -2636.29 | -2637.79 | 20.82 | -2636.06 | -2637.11 | 19.63 |
|  | run 2 | -2636.76 |  |  | -2636.43 |  |  |
|  | run 3 | -2640.33 |  |  | -2638.83 |  |  |
| UCLN | run 1 | -2637.82 | -2636.30 | 17.83 | -2637.75 | -2636.25 | 17.92 |
|  | run 2 | -2635.80 |  |  | -2635.59 |  |  |
|  | run 3 | -2635.28 |  |  | -2635.42 |  |  |
| RLC | run 1 | -2626.57 | -2627.38 | 0 | -2626.68 | -2627.29 | 0 |
|  | run 2 | -2626.92 |  |  | -2626.85 |  |  |
|  | run 3 | -2628.66 |  |  | -2628.35 |  |  |

Table S2: Clock model comparisons. Log marginal likelihood (lmL) estimates by stepping-stone sampling (SS) and path sampling (PS) and natural log Bayes factors (BF).

The BF was calculated using the following formula:

$$BF=2[ln(mL\left( model1 \right)-\ln\left( mL\left( model2 \right) \right)]$$

The model with the highest lmL was used as model 1, i.e., the RLC model. According to Kass and Raftery (1995), model 1 is favored over model 2 for BF values greater than 2 and model 2 is favored over model 1 for values smaller than -2.

Reference : Kass, R. E.  and A. E. Raftery. Bayes factors. 1995. Journal of the American Statistical Association 90(430): 773– 795.
