# Supplementary figures and images for: Recent range expansion of an intermediate host for animal schistosome parasites in the Indo-Australian Archipelago: phylogeography of the freshwater gastropod Indoplanorbis exustus in South and Southeast Asia
Source: Parasit Vectors. 2017 Mar 6;10:126. doi: 10.1186/s13071-017-2043-6 (PMC5339995; doi:10.1186/s13071-017-2043-6)

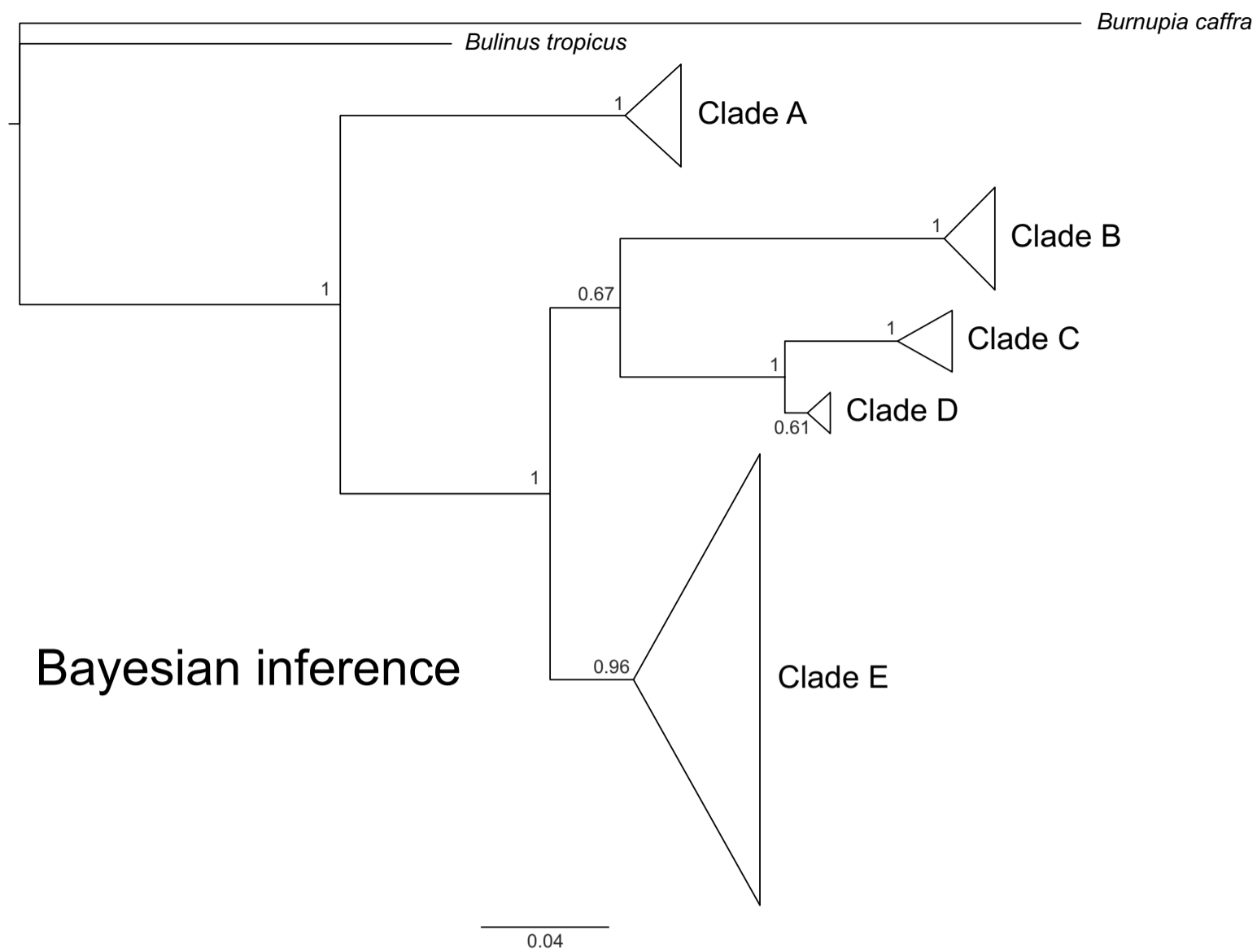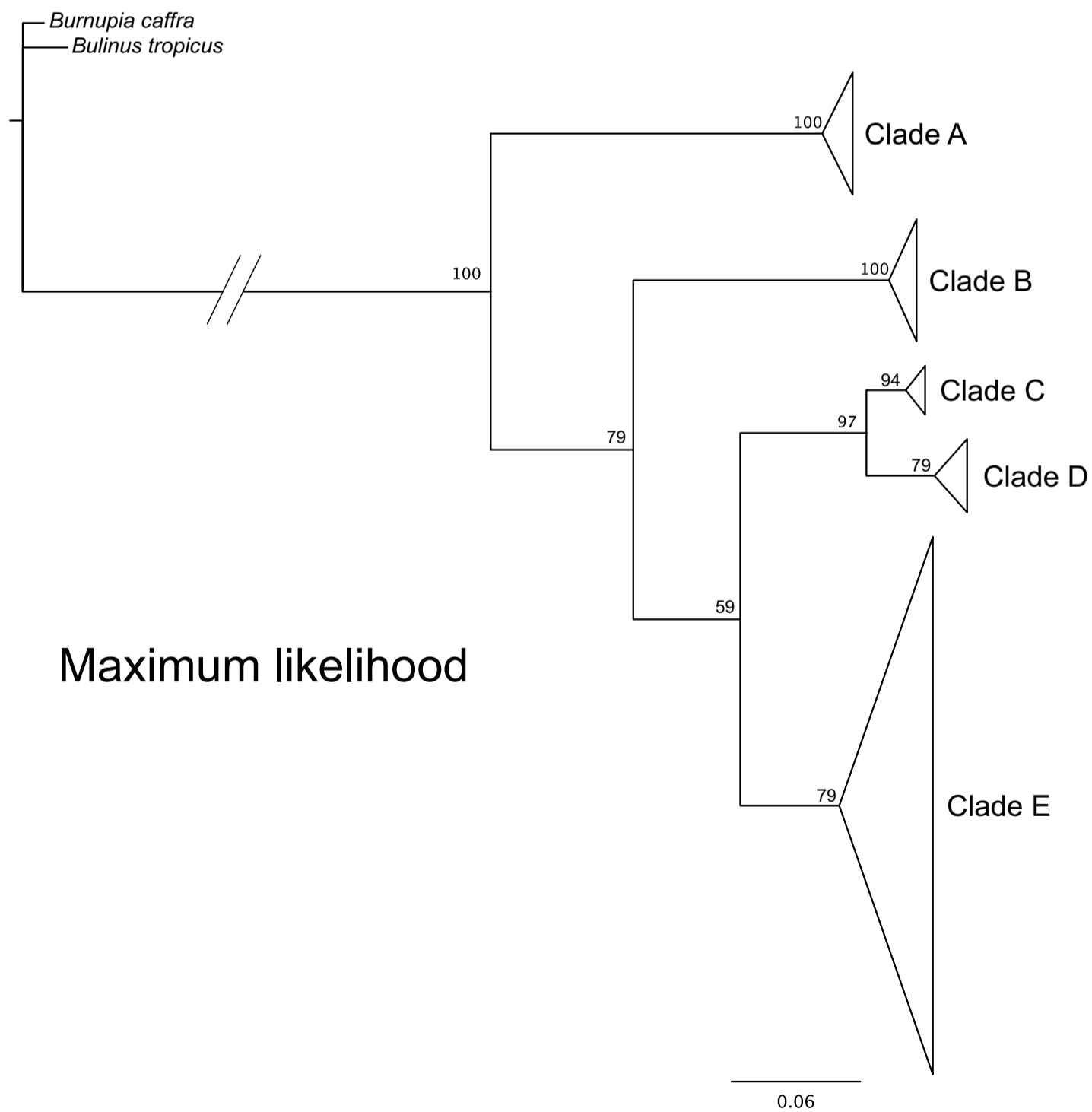

Supplement: Additional file 3: Figure S1. — Phylogeny of Indoplanorbis exustus estimated by Bayesian inference and maximum likelihood. (PDF 19 kb) [file 13071_2017_2043_MOESM3_ESM.pdf]
